# Thirty-Ninth Annual Meeting of the Mississippi Valley Association of Dental Surgeons

**Published:** 1883-04

**Authors:** E. G. Betty


### PROCEEDINGS.

### Thirty-Ninth Annual Meeting of the Mississippi Valley Association of Dental Surgeons.

REPORTED BY E. G. BETTY, D.D.S.

The Association met in annual session in the lecture room of the Dental College at 10 o'clock A.M., Wednesday, March 7th, 1883. The members were promptly called to order by President Clyde, and prayer was offered by Dr. Jerry Robinson, of Jackson, Michigan. The minutes of last year's meeting were read by the Secretary, Dr. Hoff, and no objections being made, they stood approved as read On motion of Dr. Hunter, the time of meeting for the several sessions was fixed at 9 to 12; 2 to 5:30; and 7:30 to adjournment.

DR. H. A. SMITH, Chairman of the Executive Committee, reported the following programme of subjects for general discussion:

1. Nervous and Muscular Affections dependent on Dental Irritation.
2. Etiology and Pathology of Dental Caries. Treatment by filling.
3. Periodontitis and Alveolar Abscess, pathology and treatment.
4. Prosthetic Dentistry: Restoration of features and expression how best accomplished.
5. Fitting Artificial Crowns to Roots of Natural Teeth: New and Old Methods.
6. Reports of cases in practice.

The report was, on motion, adopted.

A motion was carried to set apart the hour of 11 o'clock A.M., March 8th, to enable Dr. W. S. Howe, of Philadelphia, to exhibit some new and interesting methods in Mechanical Dentistry, and the mounting of artificial crowns upon roots.

The Committee on Publication, through Dr. J. Taft, reported that the papers and proceedings of last year's meeting were duly published in the journals, the REGISTER making a complete report of the discussions.

A recess of five minutes was had to enable the Treasurer to collect dues from the members.

DR. J. TAFT, Chairman of the Committee to draft resolutions expressing the sense of the Association upon the death of Prof. Jas. Taylor, made report:

IN MEMORIAM.*

Since the last meeting of this body one of its prominent members—Dr. James Taylor of Cincinnati, has passed away. This event produced more than an ordinary impression, not only upon the community in which he lived, but especially upon the profession of which he was an honored member. In a very marked manner is his loss felt by this Society in the organization of which he was one of the leaders.

He was a pioneer in his profession, for the West and South, indeed, his influence was felt throughout this entire country. In the early part of his career, he heartily and earnestly engaged in various enterprises for the development and progress of his chosen profession employing his time, energy and money for the purpose.

It is not possible, however, to mention all that he did. Justice, however, requires that these things should not be wholly passed by. He early took a stand for high professional culture and attainments, and not only in theory, but he early put into practice, the principle he avowed. He was one of the earliest editors of dental periodical literature. He established, and for many years edited, the Dental Register, the oldest Dental Journal with one exception in the world.

He was largely instrumental in the organization of this Society. He was mainly instrumental in the establishment of the Ohio

*This was written at the close of the meeting of 1882, but was not read at that time.

College of Dental Surgery, and was an officer or teacher from its organization till his death.

He took an interest in everything pertaining to public welfare. He was honored and beloved by all who knew him. In some respects his place will never be filled. The most befitting tribute we can pay to his memory, is to imitate to our best ability the sterling qualities that animated and guided him in all his course of life.

Your Committee suggest that this expression of regard and appreciation be engrossed upon a memorial page of the proceedings of this Society; and also that a copy in proper form be sent to her who was the greatest sufferer in this bereavement.

J. TAFT.
JAS. LESLIE.

DR. G. W. KEELY said that thirty-five years ago, when he began the practice of dentistry, Dr. Taylor was the first man in the profession to give him any encouragement. He had often met Dr. Taylor at the Society, he did more than any one else to establish this Association upon a firm footing; he worked hard and earnestly to make it a success, and with what result we all know.

DR. J. TAFT.—The report of the Committee could have been much longer. Some thirty-five years ago Dr. Taylor assisted me in my profession, and I have no doubt but that his advice has influenced my subsequent career by broadening my views and stimulating me in the right direction. Dr. Taylor always helped young men and never failed to do what he could to put them in the right way of becoming successful and useful in their profession.

DR. BERRY.—I heartily endorse all that Prof. Taft has said about Dr. Taylor. I remember that many years ago Dr. Taylor introduced Dr. Taft as a member of this Association, and that he enjoyed a peculiar pleasure in so doing.

DR. H. A. SMITH.—When I first had the pleasure of meeting Dr. Taylor, it was upon the occasion of my entering the college. I called at Dr. Taylor's office on the morning of that day, and was there impressed with the metropolitan air a busy office acquires. My whole professional career has been benefited by the advice and counsel Dr. Taylor gave me. He was a man of singularly cool, clear judgment, and measured a man correctly upon first seeing him.

Dr. Osmond. I heartly agree with all that has been said by the gentlemen preceding me. I have known Dr. Taylor for many years, often visiting his office and receiving many valuable suggestions about dental matters. Many things that we now call new can be referred back as originating with Dr. Taylor, such as treatment of dental diseases and methods of filling; just as, when we think we have come upon a new thought or saying, we will upon hunting it up, find it in Shakespeare.

Dr. C. R. Taft.—When I was a student in 1850–51, I had an aching tooth that needed filling. Upon excavating it, Dr. Taylor came upon a live nerve. He went out into the laboratory and made a small concave cap of gold which he placed over the pulp, and then filled the tooth with gold. The tooth and its filling are still doing good service, and though I'm not certain, I think it is yet alive. Can any one do better than that to-day? I apprehend that we are not so far in advance to-day of what Dr. Taylor was many years ago.

Dr. J. Taft.—It is impossible for one man to make a complete biography of Dr. Taylor, and I cordially invite all the older members of the profession to assist me in the matter by giving me such items and reminiscences as they may possess. I do this because we all want a good biography of Dr. Taylor and such an one I intend to publish in the Register. I have a biography prepared and have delayed it in order to procure a steel-plate, which can probably be obtained, when the engraved portrait will appear with the biography.

The report was adopted by the Association on a rising vote.

On motion, the Committee on Constitution and By-Laws was granted further time.

The debates on the programme being next in order, the first subject, "Nervous and Muscular Affections dependent on Dental Irritation," was announced.

Dr. Berry.—I call upon Dr. Osmond to make the opening speech.

Dr. Osmond.—Nix come arous.

Dr. Kempton.—This subject has been agitated in the journals for several years past. In investigating the subject we must be careful to distinguish between an effect and a coincidence. Persons may suffer from nervous troubles that have no connection with the teeth.

There are many instances in which other affections are traceable to the teeth, as for instance, pain in the ears. A young man once presented himself with severe pain in the ear, and but little in the tooth, the latter was extracted while a marked exacerbation of the pain in the ear almost immediately followed, finally disappearing. Dr. Sexton, an Opthalmologist and Aurist of New York, mentions many cases where the ears have been treated without success, until the dental lesions had been removed. He also cites cases of diseases of the eyes due to dental lesions. I have seen one or two cases of marked congestion of the conjuctiva connected with diseased teeth. Dr. Sexton said that of 6,000 cases he had treated about 50 per cent. were caused by dental lesions.

Dr. C. R. Taft, here introduced Dr. L. Buffett, of Cleveland, to whom was accorded the privilege of the floor; the same courtesy being also extended to Dr. J. A. Robinson, of Jackson, Michigan.

Dr. L. Buffett thanked the Association, and remarked that he was hardly prepared to discuss the subject under consideration, without having had time to think it over a little. The muscular and nervous systems are so intimately connected, that the cause of muscular troubles must be sought for in the nervous. Upon the nerves themselves we may have tremors. Again, the nerves may be either atrophied or hypertrophied.

Hypertrophy of the nervous tissues causes an exalted degree of pain and sensibility in the part to which the nerve is distributed. Atrophy sometimes produces pain, but most usually, a cessation of pain and sensibility in the periphery, and sometimes paralysis of muscular tissue.

The subject under consideration is the most difficult one to manage in the practice of our profession. In the majority of cases a hyper-sensitive condition of nerve is the cause of many troubles and pains we have from the teeth. There is never a case of nerve irritation or neuralgia in which the nerve suffers from a lack of tonicity at the point of irritation. Special paralysis and muscular atrophy upon investigation will be found to be due to the irritation of some part of the nervous system, and reflected to the periphery. Owing to the great ramification of the Tri-facial nerve, we may have a more exaggerated manifestation of the pain at any one point.

Dr. Kempton.—Dr. Brown Sequard delivered two lectures in Cincinnati some time ago, and mentioned several cases, one of which is to the point, viz: A boy was seized with an epileptic convulsion every time he put his foot on the floor when getting out of bed, and though he repeatedly tried it, the same result followed. Upon examination of the foot a splinter of wood was found imbedded in the integument; it was cut away and the convulsions disappeared. The distinguished lecturer said that very slight and apparently insignificant causes were sometimes attended with serious results. In view of this it is well to have the teeth looked after when there is nerve trouble.

Dr. J. Taft.—It would be well to have some sort of a classification upon which to proceed in the discussion of this subject. For instance, the effect of reflex-action upon the nerves. Every muscle has its nerve that controls and modifies its action. The sensory nerves carry the sensation from the periphery to the muscle, in that way referring the pain to the muscle.

The hour of adjournment having arrived the discussion was postponed.

Dr. J. B. Kidd, of Lexington, Ky., was elected to membership by the Secretary's ballot on motion of Dr. Hunter.

### AFTERNOON SESSION.

The Association was called to order by President Clyde, the minutes of the morning session being read and approved. The discussion of the first subject was resumed as follows:

Dr. J. A. Robinson—I once saw a case of paralysis of the arm caused by a dental lesion. The patient was a lady *æt*

thirty-eight years, whom I first met in a physician's office. Her left arm had been paralyzed for three months. Upon examining her mouth I found the second and third molars of the left side both badly decayed. These I extracted, and the patient rapidly recovered. Another case was one of neuralgia. The gentleman experienced pain every time he stroked his beard, which was long and full. The cause seemed to be due to an upper cuspid tooth that had been filled. I prescribed a topical application of chloroform to the face, which gave him relief for a day or two. The pain returned, so I removed the filling and destroyed the remains of the nerve. This having no permanent effect I gave him heroic doses of quinine from which he obtained relief for two or three weeks, when the pain again returned. I then put him on Fowler's Solution with but little benefit. I then went back to the chloroform, and by persisting in its application the trouble finally disappeared.

Dr. G. W. Smith has had no such cases in practice. It is well to look at the different kinds of nerves there are, and observe the different effects they have. A sensory nerve produces its effect on the sensorium; the mortor nerve produces its effect on the muscle by reflex action. When a nerve trunk is cut, an irritant on the trunk end produces sensation, while the same on the periphery produces no effect, this of course is true of sensory nerves. On the motor nerve when cut, an irritant will cause contraction of the muscle to which the nerve is distributed.

Dr. H. A. Smith.—A rather novel case occurred in the College Clinic during the past winter. The patient was a healthy young man. The superior maxilla was attacked by caries. Three of the incisors were found to have dead pulps, and were hyper-sensitive. The left lateral was drilled into, and all the pain instantly disappeared. The hyper-sensitiveness must have been caused by the irritation of the dead pulp and the pain being reflected to the point usually occupied by the pulp. A sinus was made for the purpose of treatment, and forty-eight hours after the sub-maxillary gland was largely swelled. Any application of treatment was shortly followed by renewed swelling of the gland; this I mention to show how slight a degree of irritation will sometimes induce a serious inflammation. Another case was one of facial paralysis. I found upon examination that it was due to an abscessed lower tooth that had been filled with amalgam. The filling was removed and the abscess yielding to treatment, the case gradually recovered.

DR. KEMPTON.—The importance of this condition varies with the kind of case. When the dental lesion is insignificant and the local marked, the cause may be overlooked, and the disease treated without favorable results to the prejudice of the patient. It is important, then, that the teeth should be examined in all obscure cases. In other instances the dental lesion is so marked that attention is immediately drawn to the teeth. In one case of facial neuralgia the teeth were all more or less carious, and some twenty odd were extracted. The neuralgia disappeared. In this case there had been no pain in the teeth, and in such instances the cause is apt to be overlooked.

DR. SAGE saw a case of St. Vitus' dance in a boy aged 12 years. The physician's treatment was palliative, though he advised the extraction of a diseased lower molar. As the patient was in bed, he was chloroformed, and examination showed a dead molar with incipient abscess. This was treated, and the patient recovered.

DR. KEMPTON.—A German journal published an account of a case in which the toothache was violent, though the affected tooth was sound. Upon cutting into it, calcific deposits were found in the pulp chamber. The nerve was killed, and canal filled. This had to be done for a number of teeth though all had to be extracted before final relief came.

DR. OSMOND cited a case of a boy aged fourteen years, who had suffered severely with neuralgia arising from a bicuspid tooth. Treatment benefited the patient somewhat, though permanent relief could only be had from extraction. On splitting open the tooth, a nodule was found in the pulp which I still have in alcohol.

DR. G. W. SMITH cited a case in which he had discovered a number of nodules in the pulps of teeth in a patient *æt.* 25. Dr. Smith was at this time elected a member of the Association by the Secretary's ballot.

Prof. J. Taft.—I am scarcely prepared to speak on this subject, at this moment, as it is a very broad one, and one that requires thought and careful consideration. It is one that needs for its treatment a long and intelligent experience upon which to base argument. If there is pressure upon a nerve there will be one of several manifestations: paralysis, neuralgia, or inflammation. The slightest cutting or wounding of the finger is oftentimes the cause of so serious a consequence as tetanus. The administration of violent poisons—those acting very suddenly—will produce a perfect interruption of nerve function so marked as to destroy it altogether. *How* it is done we do not know. In first dentition, disturbances are produced by an irritation reflected to the pneumogastric nerve. In retarded first dentation, convulsions arise in many instances, being a contraction of the muscles, sudden and violent. I do not think that the disturbances accompanying first dentition are caused by a pressure of the erupting teeth upon the over-lying tissues. It is more likely due to a lack of nutrition to the parts of the nervous system involved.

Dr. Buffett.—Is there not pressure upon some nerve in some part of the nervous system?

Dr. J. Taft.—The pressure may exist and possibly does, but we cannot detect it as it is so slight. In malarious regions of the country, neuralgias are prolific, but we cannot say that they are caused by pressure upon some nerve.

Dr. Buffett.—I believe we always have pressure where there is pain. Even if we cannot find it, it is reasonable to suppose that it does exist, and I repeat, pressure always exists where there is pain.

Dr. Betty.—If the theory that pressure produces the pain is correct, why is nerve-stretching resorted to?

Dr. Buffett.—To relieve the pressure.

Dr. Betty.—Is not excision or a removal of a part of the nerve effectual?

Dr. Buffett.—Extension is only resorted to after excision.

Dr. Howe.—In every motion we make, matter goes through a process of destruction or metamorphosis, and so long as the

"'street-cleaning department" * carries it off, all right; but should there be more effete matter formed than can be carried off, pain will result.

PROF. J. TAFT.—If the nourishment of a nerve is reduced in amount, the nerve will suffer; I don't say, pain, but it suffers nevertheless.

DR. BUFFETT.—You may have atrophy of a nerve, but there will also be found pressure at some other point along the course of the nerve.

DR. TAFT.—That is an open question, and I think it conveys a false impression to say that all pain in a nerve is due to pressure.

DR. SAGE.—The swelling of glands is due to a sympathetic action of the nerves, as for instance, the swelling of the glands in the groin in gonorrhoea. In specific diseases, the glands far remote from the local trouble exhibit the swelling and inflammamation.

DR. CLYDE mentioned a case of "milk crust," and wanted to know if that was a result of dental lesion.

DR. OSMOND. I, too, have seen eruptions upon the scalp and skin of infants, and have relieved the whole thing by simply scarifying the gums.

DR. J. TAFT.—We must not infer that all scalp diseases owe their origin to some dental lesion, though there are undoubtedly cases whose cause may be found in the teeth and dentition.

DR. WRIGHT. In South Germany and Switzerland the people seem to be superstitious on the subject of scabby heads. It is a common thing for the parents to foster the scabby head by covering it with a cap of some kind, never disturbing it, and leaving the scabs to thrive underneath.

DR. KEMPTON. This scabby head, or "milk-crust," is most common among the Germans, and is probably due to the kind of food that is consumed, an inferior quality of which, together with a great deal of beer, to say nothing of a dearth of soap, being enough to produce almost anything.

* Dr. Howe being an old Cincinnatian; and the subject of street-cleaning being much agitated of late, he may be pardoned the use of this expression.—REP.

Dr. Buffet. The teeth have nothing whatever to do with eruptions upon the scalp, further than that a disturbance in any part of the system favors this other process in the scalp by weakening the tone of the system. In my opinion, these infantile scalp diseases are the result of the severe washings the new-born infant is subjected to. I regard it as very fool-hardy, and I direct that they shall be thoroughly annointed and then merely wiped off.

Dr. Kempton. During my experience as a medical practitioner, I have always instructed the nurse to wash the child thoroughly. The oil or lard used in annointing forms a kind of emulsion with the sebaceous matter on the surface, and is easily removed.

Dr. J. Taft mentioned five cases of nerve-stretching, four of which were successful, the fifth being a failure. I was not aware, until Dr. Buffett told us, that extension of the nerve was only resorted to *after* exsection."

Upon motion the subject was passed; the second being announced for discussion. Dr. G. W. Smith read part of a paper bearing upon the subject. The point of his paper was a description of a paste for capping exposed pulps and is as follows:

R, Ol Anisi ʒI.

Creosote (wood) ʒII.

These are mixed together in a bottle, and are kept tightly corked. The powder is composed of Zinc oxid and Sodæ Biborate, each an ounce, well mixed together. When the capping is needed, the liquid and powder are made into a paste, which is applied right over the pulp, and the filling put over that.

Dr. J. A. Robinson.—I have been in the habit of making fillings in sections as it were. After the capping has set, I trim it away from the cervical border, and put in about one-third of the permanent filling at that point. I then remove the surplus from the upper portion of the capping, and introduce the remainder of the filling.

The philosophy of the method is to prevent the capping from changing its original position, while under the strain incident upon the introduction of the filling: When the oxy-chloride is placed over the paste, or covering directly in contact with the pulp, the attraction of the chloride will lift the application away, leaving a space under the capping between it and the pulp. This I can readily prove by making a capping on a piece of glass from the under side of which, the space I speak of, can very readily be seen.

Dr. Osmond. In capping a pulp with gutta-percha dissolved in chloroform, the evaporation of the latter causes the gutta-percha to draw away and leave a space between it and the pulp.

Dr. Smith (G. W.) said that all vegetable oils are easily absorbed, and that his method saved the teeth. No matter *how* it was done, the fact spoke for itself.

Dr. Kempton.—As part of the subject is "treatment by filling," I would like to say that the oxyphosphate of zinc has a large field before it. I have in some cases seen it withstand the wear of two years time. Adjourned.

### Second Day, Thursday, March 8th.

### MORNING SESSION.

The Association was called to order, by President Clyde, and the minutes of the previous session read and approved. The discussion of the second subject on the programme was resumed.

Prof. J. Taft.—This subject is one of the very large ones, and there is material sufficient in it to furnish discussions for two or three days. In regard to the causes of dental decay, there is so much extraneous matter thrown in, and so many theories elaborated, that the whole subject appears to be greatly confused. When you ask a man his views upon dental decay, he will answer "Oh! I dont know anything about it." There are a number of things concerning dental decay, that are well fixed and definitely understood. Opinions upon all subjects, of course, differ; as for instance you ask one man, what is an element? He will say "oxygen;" another will tell you "he does not know." So it is regarding this subject; since there are so many conflicting views and theories, we must take a stand and find out just what we have attained. We know of what the teeth are composed, and that one class of the constituents is inorganic, and the other organic.

We also know that the teeth possess vitality. A prominent man in the profession some years ago maintained that the dentine possessed no vitality, and especially the enamel. His theory was that the teeth are mineral, but he has now changed his views. After the teeth are formed, they undergo some changes; for instance, their density increases. Now what can it be that makes this change, if it is not vitality? In the order of development, the organic structure is first built up, and the inorganic is afterwards supplied; this we know and understand very well.

Now as regards decay: the inorganic is first taken away, leaving more or less of the organic. The varieties of decay differ, presenting different aspects. In some varieties the organic and inorganic constituents both disappear; in others, the one or the other may be left in greater or lesser quantity.

In the dark decay, the structure is broken down, but both constituents remain, and that fact probably prompted the statement of Prof. Mayer, that "in the dark decay he found a large portion of the inorganic constituents remaining."

In some forms of decay, the organic portion retains its form in a marked degree. In other forms the organic structure is wholly broken up, when the inorganic portion is removed. There are agents that produce decay of the teeth, or will dissolve the organized phosphates, and the question naturally arises "can these agents—these acids be found in the mouth?" That question, however, has been ably settled to the satisfaction of all. These acids *are* formed in the mouth, and when we find that the inorganic parts have been removed, we are pretty well satisfied that there must have been an agent to produce this effect. A new theory is being promulgated to account for the whole process of decay—that is very confusing to the young men just entering the profession. Many of the new theories of Pasteur, Koch, and others, still require additional proof to fully establish all that is claimed for them. There are parasitic growths of various kinds, and the question is, how far are they concerned or instrumental in the process of decay. One theory is that these organism exude from their bodies a fluid that dissolves or decays the tooth substance and structure. Young men are far more likely to become confused with such theories as these than are men of ripe experience and judgment.

Decay of the teeth is not a pathological process. A pathological process can only take place in a living structure. In tooth decay, the pathological occurs before devitalization, and then the decomposition supervenes.

The statement that dental decay is a pathological process is misleading and should be corrected. In most decays of the teeth, the inorganic constituents are first removed, leaving the organic, and it is in the residuum of the organic that these parasitic growths are found to exist. They cannot exist in the tooth before decomposition takes place. Parasitic growths in other parts of the body exist as secondary matter.

DR. H. A. SMITH.—In the nitric acid decay, when the inorganic portion is being destroyed, I would like to know if there is sufficient organic matter in the exposed layer to generate enough acid to produce a progressive decay exclusive of the introduction of putrefactive matter from the outside?

DR. J. TAFT.—Before caries occurs there must be devitalization in the part.

DR. WRIGHT.—I have been very much interested in Prof. Taft's remarks and especially do I agree with him in that there is no *real* conflict in the theories of decay, it being as he says, more confusion than conflict. I think that the several theories are merely different phases of the same question, and that they should all be gathered together under one banner

For instance, inflammation is an abnormal process, in which there are a great number of distinct changes in the elements in the capillaries and larger blood-vessels. Changes in the blood, in its lymph, in the nerve action and function of the part. The symptoms—pain, heat, swelling and redness—simply attend upon these changes. Taking the whole disease of dental caries, I think there are pathological processes that take place.

It is not especially necessary to discuss the formation of acids and ferments in the mouth. I think there is a degree of inflammation preceeding. The disease, dental caries, and all the phases of the acid theory, bacteria, bacilli, micrococci, sprosspilz, etc., and all other facts concerning dental caries, are but parts of one general disease, we each seeing but one phase.

Dr. J. Taft.—In the incipiency of dental decay, pathological processes take place, but not after devitalization.

Dr. Wright.—Cannot these pathological changes be only one part or phase of the whole question of dental caries?

Dr. J. Taft.—It is wise to discriminate and not become confused when considering this matter. We cannot say with truth that a dead man is in a pathological condition.

Dr. Wright.—Then, Dr. Taft does not regard decay of the teeth a disease?

Dr. J. Taft.—I do not. Of course, caries is preceded by disease. As the process goes on, there is a line of demarcation between dead and living. In the dead part, there can be no disease, while there may be in the living, and while the process goes on, the line of demarcation is moving onward.

Dr. Wright.—By your definition, caries is not a disease, but a result of disease.

Dr. J. Taft.—It follows. We have failed in this matter, and have groped along all the while. We give too many names for the same thing.

Dr. Wright.—I at first agreed with Dr. Taft, but now I disagree. He at first said caries was a disease, and afterwards said it was but a result of disease.

Dr. J. Taft.—The only difference between us is the shading of the definition.

Dr. H. A. Smith.—Dr. Taft says death of a portion promotes decay by the putrefaction of that part furnishing solvents. Any change that takes place in a tissue is pathological. Does the putrefaction of the organic matter of the tooth produce sufficient acid to destroy all the inorganic part of the tooth?

Though I have been brought up on the acid theory, and still cling to it, yet I do not regard it as thoroughly established, and believe both it and the bacteria theory to be hypothetical.

Dr. Wright.—I think that all these processes are but parts of this whole question of disease, and until we take a comprehensive view of it and include them all, we shall still be at sea and no further ahead than we were forty years ago.

The Association granted Dr. J. A. Robinson sufficient time to read a paper. Dr. Porre also read a paper. Eleven o'clock having arrived, (it having been made a special order of the business,) Dr. W. S. Howe, of Philadelphia, made a detailed demonstration of his method of mounting artificial crowns upon roots in the mouth.

AFTERNOON SESSION.

The Association was called to order, Pres. Clyde in the Chair. The minutes of the morning session were read and approved. On motion of Dr. J. Taft, a Committe of three was appointed to draft a suitable memorial upon the death of Dr. W. H. Goddard, of Louisville, an old and early member of this Society. The Chair appointed on this Committee, Drs. J. Taft, J. S. Cassidy, R. J. Porre. The Treasurer now made his report, which was referred to an Auditing Committee.

The discussion of the second subject on the programme was resumed.

Dr. J Taft.—I think it is a good idea to determine at what period or periods caries is most likely to occur. During any condition of enfeeblement, the secretions of the mouth become vitiated, and the teeth in consequence more prone to decay. During the periods of gestation and lactation, they are less perfectly nourished, and necessarily become more susceptible to the influence of decaying agents. The largest proportion of decay of the teeth, takes place at night, during the hours of rest. Throughout the day the secretions are most abundant, and the parts about the teeth are kept in motion in the acts of speaking and masticating. At night the organs are quiet, and the secretions in abeyance. Many persons acquire the habit of sleeping with the mouth open — the passing of air through it dries up the moisture, agglutinates the mucous, thus making the opportunity more favorable for putrefaction. In the morning there will be found in the mouth a "bad taste," this being caused by the vitiation of the secretions. When kept closed during the night, these ill effects do not appear, and the mouth will taste fresh and clean. The agglutination of the mucous and its subsequent decomposition are the cause of the offensive taste and breath, to say nothing of the deleterious results to the teeth. I

have given this subject some attention for some years past. Everything being equal, the mouth when kept closed will be in a better condition than when is habitually kept open.

Dr. Osmond. The inference then is, that those who snore must of necessity have the mouth open, and in consequence have bad teeth. The ages between thirteen and twenty years, is the period of greatest ravages by decay, especially in those whose growth has been rapid. With girls, the most critical period is during the establishment of menstruations and more so if rapid growth occurs at the same time.

Dr. F. A. Hunter.—I am very glad indeed that this subject was so enlarged upon the programme, as to include the capping and treatment of exposed pulps. I have spent a great deal of time experimenting upon the subject, and with a most marked degree of success. I do not hesitate to say, and I say it boldly and without fear of contradiction that, by my method, I have succeeded in saving every exposed pulp that has come my way; yes, even though the pulp may be suppurating and the pus welling up in volumes, I shall save it. I can save even *remnants* of pulps, be they never so small.

Some years ago—I can't just recall how long; at any rate, it was about the time of the introduction in our city of the English sparrow, I began experimenting with a capping. It is so good and efficacious that your patients will bless you; you become happy in consequence, they leave your office showering down upon you the highest enconiums, and—never come back again. Some one remarked during the course of the discussion, not to make pressure upon the pulp. Now, I say that pressure is just the very element upon which a great deal of the success depends. Drive your capping down as hard as you can, and, as Siddal says, "You have the pulp in a tight place." Now, as to the composition and mixing of my infallible capping. It——

Voice. Write it on the board.

Dr. Hunter. That is probably best, as many, no doubt, will wish to experiment for themselves, and here it is:

℞ Sorgum Molassum, 1 pint.
Droppum English Sparrow, 1 pound.

Mix into a stiff plastic mass and apply. I must confess that though my success has been fully equal to 98 per cent. of all cases with this capping, I have never experimented with chickens. I have no doubt but that their droppings are equally advantageous." Dr. Hunter here took his seat amid storms of applause.

Dr. G. W. Smith advocated cleanliness.

Dr. Porre. I am nothing, if not practical. I care nothing for theory. The air is loaded with bacteria and other bugs and their injurious effects in the mouth can be neutralized by cleansing the mouth at night with an antiseptic.

Dr. Howe. A little close observation will show the gentle snoring to be the hum of the bacteria, and the boisterous form to be the roar of the leptothrix.

A motion was made to pass the subject and take up the third, which was done. Dr. G. W. Smith favored the members with that half of his paper which bore on *this* subject.

Dr. Sage thinks that dentists have too much neglected the free use of the lance during the incipient stage of an inflammation in the alveolus. At a later period it will not avail, owing to the morbid deposits that take place. I do not think an alveolar abscess is ever thoroughly cured, and I base my statement upon the surgical axiom that a part once inflamed is always ever after more likely to have a recurrence of the inflammation.

Dr. H. A. Smith. Dr. Chas. Tomes, in a paper recently published in the *New England Dental Journal*, advocates the filling of the pulp cavity with some antiseptic matter, so that when the canal is opened with a broach, no micro-organisms in the atmosphere can find access to the socket through the root. I think that we should always avoid introducing septic matter through the apex of the root, and endeavor, if possible, to exclude micro-organisms and the production of putrefaction. Again, if the bacteria theory is true, the putrefaction in a tooth devitalized by a blow can only receive the bacteria through the circulatory system.

Dr. J. Taft. While there is some truth in the theories that have been canvassed to-day, there is a disposition to go too far. Some years ago Virchow promulgated his theory of cellular pathology. Things have so changed since, that he has been obliged to revise and modify his former views. His new one is an entirely different work. When a tooth is devitalized, the tissue is given up to simply chemical changes, and in this organisms have but little play.

Dr. Wright. We, as dentists, have but two diseases to treat, viz: inflammation and dental caries. These are the cause of all our contests, discussions and disagreements.

THIRD DAY—MORNING SESSION.

The Association met promptly and was called to order by President Clyde. The minutes of the previous session were read, and there being no objections, they stood approved. A motion was carried to pass the third subject, and the fourth subject, "Prosthetic Dentistry; Restoration of Features and Expression How best Accomplished," announced.

Dr. Berry. Prosthetic dentistry, or *mechanical* dentistry, as we have many years been in the habit of calling it, has received but little attention in this Society for several years past. The restoration of features, etc., is best accomplished by restoring so far as may be, the order of things as they existed before the loss of the dental organs. Nature usually does her work well, and we cannot do better than model our work after her design. The secret of John Allen's success as a prosthetic dentist is his habit of carefully studying each case that comes under his notice. By learning them thoroughly he has been enabled to restore feature and contour with great success.

Dr. G. W. Smith arose to his feet and began to talk, but as his remarks were irrelevant, it was only after being called to order three or four times, that he finally got started in the neighborhood of the right direction. He said that there are many ways of restoring the features, and not least important was the harmonizing of the color of the teeth with the complexion of the person. We should also study the contour of the face in all its varieties of length, breadth, etc., and adapt the denture accordingly.

Dr. Wright. If I should wish to restore the expression of those around me here that are wearing artificial teeth, I should certainly remove their plates. [Laughter.]

Prosthetic dentistry has been dragged in the dust by the introduction and use of block teeth. In the old time, when ivory, the teeth of dead subjects, sheep, calves, etc., were used to replace the human teeth, the effect was certainly more artistic than that now accomplished by the use of block teeth.

Dr. Kempton. My weak point is prosthetic dentistry. I find that it is next to impossible to get teeth of the proper color and shading.

Dr. Wright. In block teeth the color is too uniform and "set" throughout, such as is not seen in the natural organs. It is much better to grind off the enamel of the machine teeth, and modify the color by staining and re-enameling.

Dr. Berry. Some years ago there was a use for the teeth that were extracted from the mouths of dead subjects. They were riveted on plates and mounted on pivots on the roots.

Dr. Osmond. Artificial teeth, when polished instead of enameled acquire that peculiar bony surface, which is far more natural than the staring gloss of enamel.

Dr. Porre. Who is responsible for the manufacture of machine teeth?

Dr. Wright. The dentists.

Dr. Porre. Dentists should send models and have the teeth made to suit the case.

Dr. H. A. Smith. Dr. Howe exhibited yesterday a number of sets of rejected teeth. These were irregular in many slight ways, and some of the irregularities were very natural looking indeed. But these sets do not sell well, as most dentists prefer to have them made as regular and uniform as possible. We don't attempt to restore features, though we may know how, neither do we expend upon the case sufficient time to study all its varieties and peculiarities of contour, expression, etc. This, I imagine, is because we work for a uniform fee and guage the work accordingly. We, ourselves, are to blame for our poor attempts at prosthetic dentistry, and should not shift the blame upon the manufacturer.

Dr. Wright. It is as much the fault of the manufacturer as it is of the dentist.

Dr. G. W. Keely. The restoration of contour is an exceedingly important thing, and right here let me say that the profession is more indebted to John Allen than to any other man. He first made use of plumpers to restore the contour of the face. Continuous gum offers great facility for the restoration of contour: it may also be done with rubber, but there can be no comparison between the kind and quality of the work. The dentist who does not consider the restoration of contour does not do his duty. The pivot teeth of old Dr. Stockton were better and stronger than any that have ever been made since.

On motion, the subject was passed, and the Association proceeded to the election of officers, with the following result:

President.—C. M. Wright, Cincinnati.

First Vice-President.—H. A. Beamer, Cynthiana, Ky.

Second Vice-President.—C. I. Keely, Hamilton, O.

Recording Secretary.—W. H. Cameron, Cincinnati.

Treasurer.—F. A. Hunter, Cincinnati.

Corresponding Secretary.—C. R. Callahan, Hillsboro, O.

The President elect was conducted to the chair by Drs. F. A. Hunter and C. I. Keely, and upon assuming his duties, made a few appropriate remarks. On motion of Dr. Betty, the Association passed a vote of thanks to the retiring President and his assistants. Dr. J. Taft, Chairman of Committee on Memorial, made the following report:

### IN MEMORY.

Within the last week, one who was a prominent member of the profession, and from the time of its organization a member of this Society, viz: Dr. William H. Goddard, of Louisville, Ky., has passed into rest. He closed his long and useful life on Sunday, March 4th, 1883. Like a grand old oak, he had sturdily braved the tempests and chill blasts of life's varied experiences for nearly four score years. But at last he has fallen, having reached the greatest eminence in his chosen profession for which he labored with such energy and efficiency in this western country. Faithful, true and devoted in all his trusts and obligations; genial, courteous and loyal in his friendships, his excellence and worth of professional and private character were acknowledged by all.

For more than fifty years he devoted himself to the profession in which he was an untiring, sincere and painstaking worker—always to the front with those who were seeking advancement, receiving and contributing full measure in return—modest and unpretending in his relations with his professional brethren, yet manly in asserting his opinions, and bold and unswerving in defending his convictions of the right, honors sought him, as they always should the deserving, and rested gracefully on his head for fifteen years in re-election to the office of Treasurer of the American Dental Association. At the last meeting of that Society in this city, he was elected almost unanimously upon the first ballot, its chief officer—President—thus fittingly crowning his life with the highest honor in the gift of the profession.

He was one of nature's noblemen with purposes ever true and pure. As a friend, a father, and a husband he was a model worthy of all imitation. It is with great gratification that we call to memory and put upon record a few of the characteristics of our dear brother. To the bereaved family we extend our warmest sympathy in this hour of sore trouble.

Your committee recommend that a copy of this statement be entered upon a memorial page of the proceedings of this body, and that a copy in appropriate form be sent to the family. Also that a copy be sent to each of the dental journals of the country.

J. Taft,
J. S. Cassidy.
R. J. Porre.

Dr. H. A. Smith moved that the report be accepted. Dr. Betty moved an amendment to adopt by a rising vote. The amendment was accepted by Dr. Smith, and on the question being put, the members rose to their feet in silence. Drs. G. W. Keely, A. Berry, H. A. Smith and J. Taft all made obituary remarks.

The President announced the following standing committees for the ensuing year:

Executive.--J. Taft, E. G. Betty and M. H. Fletcher.

Membership.—F. A. Hunter, C. I. Keely and A. G. Rose.

Publication.—E. G. Betty, J. M. Clyde and O. N. Heise.

Ethics.—G. W. Keely, H. A. Smith and J. G. Cameron.

After the transaction of miscellaneous business, the Association adjourned to meet the first Wednesday in March, 1884.

